# Petromylidenes A–C: 2-Alkylidene Bile Salt Derivatives Isolated from Sea Lamprey (*Petromyzon marinus*)

**DOI:** 10.3390/md16090308

**Published:** 2018-09-01

**Authors:** Ke Li, Anne M. Scott, Skye D. Fissette, Tyler J. Buchinger, Joseph J. Riedy, Weiming Li

**Affiliations:** Department of Fisheries and Wildlife, Michigan State University, East Lansing, MI 48824, USA; like4@msu.edu (K.L.); Scottan7@msu.edu (A.M.S.); fissette@msu.edu (S.D.F.); buching6@msu.edu (T.J.B.); riedyjos@msu.edu (J.J.R.)

**Keywords:** bile salts, pheromone, electro-olfactogram, behavioral assay, invasive species, cyclostomata

## Abstract

Three novel bile acid derivatives, petromylidenes A–C (**1**–**3**), featuring uncommon alkylidene adductive scaffolds, were isolated from water conditioned with sexually mature male sea lampreys (*Petromyzon marinus*). Their structures were elucidated by mass spectrometry and NMR spectroscopy, and by comparison to spectral data of related structures. The identification of compounds **1**–**3**, further illustrates the structural diversity of the 5α bile salt family. Compounds **1**–**3** exhibited notable biological properties as well, including high olfactory potencies in adult sea lampreys and strong behavioral attraction of ovulated female sea lampreys. Electro-olfactogram recordings indicated that the limit of detection for **1** was 10^−9^ M, **2** was 10^−11^ M, and **3** was less than 10^−13^ M. These results suggested **1**–**3** were likely male pheromones, which guide reproductive behaviors in the sea lamprey.

## 1. Introduction

**Figure 1:**
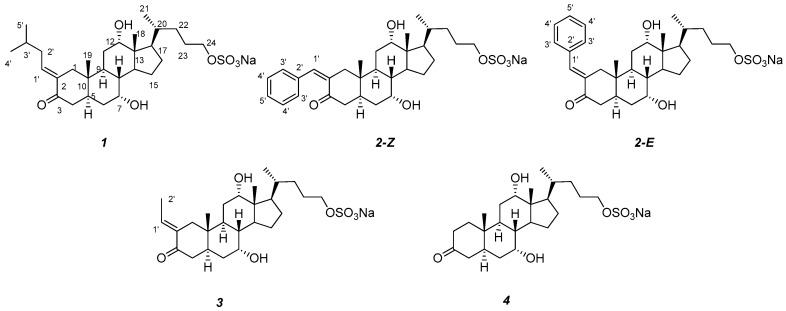
Structures of petromylidenes A (**1**), B (**2**), C (**3**), and 3kPZS (**4**).

The sea lamprey is an invasive, parasitic vertebrate in the Laurentian Great Lakes. One emerging option to control the invasive sea lamprey population is the application of the pheromones, to modulate reproductive behaviors in this species [1]. Investigations of pheromone communication in sea lamprey have unveiled that sea lampreys, similar to many other fishes, have an olfactory system which is acutely sensitive to bile salts [2,3]. Furthermore, spermiated male lampreys (sexually mature males with expressible milt) produce unique sulfated bile alcohols with a C24 backbone and 5*α* configuration, which aggregate potential mates during spawning [4,5,6,7,8]. In our ongoing investigation for structurally diverse and biologically active compounds released from spawning male sea lampreys, we identified three novel bile salts trivially named petromylidenes A–C (**1**–**3**, Figure 1), and a known compound 3-keto petromyzonol sulfate, 3kPZS (**4**, Figure 1). Petromylidenes A–C (**1**–**3**) possess the same steroid framework as the main compound already described 3kPZS (**4**) but differ from each other in the nature of the alkylidene substituent at C-2. The double bond geometry in the alkylidene moiety was assigned as *E* in **1** and **3**, whereas **2** was a mixture of both *E* and *Z* isomers. Here, we describe their isolation from water conditioned with spermiated male sea lampreys, structure elucidation, and biological activity assays.

## 2. Results and Discussion

**Figure 2:**
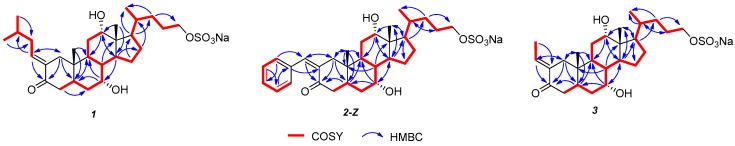
Key COSY and HMBC correlations of petromylidenes A–C (**1**–**3**).

Petromylidene A (**1**) was obtained as a yellowish oil with [α]${}_{D}^{25}$ +6.6 (c 0.10, MeOH) and has the molecular formula C_29_H_48_O_7_S as established by HRESIMS (*m*/*z* 539.3058 [M−H]^−^), which indicated six degrees of unsaturation. The ^1^H and ^13^C NMR spectra (Supplementary Materials Figures S1 and S2) revealed that **1** has a close resemblance to 3kPZS (**4**) [9], and further confirmed a minor modification on ring A. Analysis of the multiplicities and the vicinal *J* values of the two carbinol methine protons in the ^1^H NMR spectrum (Table 1), allowed us to assign an equatorially-oriented plane to C-7 and C-12. Axial hydroxyl groups placed at C-7 and C-12 are consistent with the substitution pattern of sulfated steroids, established by Hoye et al. [10]. The remaining subunit C_5_H_10_ was assigned as 3-methylbutyl, based on the COSY and HMBC correlations (Figure 2). The connectivity between 3-methylbutyl and 3kPZS (**4**) skeleton at C-2, with a double bond, was supported by HMBC correlations (Figure 2) from H-1′ to C-1 and C-3, and from H-1 to C-1′. The presence of the double bond was indicated by the chemical shifts of CH-1′ (*δ*_C_ 141.8, *δ*_H_ 6.68) and C-2 (*δ*_C_ 137.3). The planar structure of **1** can be assigned as 2-(3-methylbutylidene)-3kPZS.

**Figure 3:**
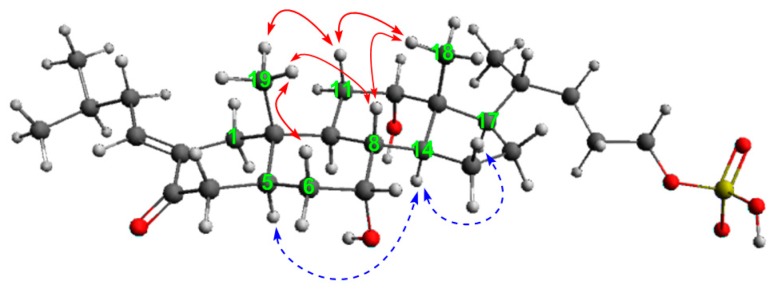
Key NOESY correlations for petromylidene A (**1**).

The NOESY key correlations of **1** (Supplementary Materials Figure S9) from H_3_-19/H-8; H-8/H_3_-18; and H_3_-18/H-20 indicated that CH_3_-18 and CH_3_-19 were on the *β* face, confirming the stereochemistry of the steroidal skeleton. In addition, the correlations from H-5/H-14, H-14/H-17 supported the orientation of H-5 and H-14 on the *α* face (Figure 3). The stereochemistry of double bond between C-1′ and C-2 was assigned by comparison of the chemical shift values of H-1′ with that of the *E* and *Z* double bond, of the model compounds. In the ^1^H NMR spectrum of **1**, the olefinic proton resonated at *δ*_H_ 6.68. The olefinic protons in *E* and *Z* configurations of analogs, yielded characteristic signals at *δ* 6.62 and 5.60, respectively [11,12,13]. The proton resonance of **1** was in good agreement with that of *E* configuration. These values were supportive of the stereochemical assignments of *E* configuration, for the double bond located on C-2. Thus, the structure of **1** was assigned as 2-(*E*)-(3-methylbutylidene)-7α,12α,24-trihydroxy-5α-cholan-3-one-24-sulfate and trivially named petromylidene A.

Petromylidene B (**2**) was obtained as a pale-colored oil with a molecular formula C_31_H_44_O_7_S, as established by HRESIMS (Supplementary Materials Figure S2). The (-)-HRESIMS at *m*/*z* 559.2729 [M−H]^−^ matched well with that of the predicted molecular formula, which indicated ten degrees of unsaturation. The ^1^H NMR and ^13^C NMR data (Table 1, Supplementary Materials Figures S10 and S11), suggested that **2** also has a 3kPZS (**4**) skeleton and a substitution on ring A at C-2. Additionally, the ^1^H and ^13^C signals of benzyl group were observed, and accounted for the additional four degrees of unsaturation in **2**, as compared to **1**. The presence of the benzylidene subsitituent on C-2 was supported by the HMBC long range correlations from H-1′ to C-1 and C-3, and from H-1 to C-1′ (Figure 2). Interestingly, the ^1^H NMR spectrum displayed two doublets at *δ*_H_ 6.47 (d, *J* = 1.9)/7.53 (d, *J* = 2.9), respectively, for the olefinic proton on C-1′. Attempts to isolate the isomers of **2** by choromatographic method were not successful. Compared with similar analogs described previously [14,15,16,17,18], the compound with olefinic proton resonace at *δ* 6.47 (d, *J* = 1.9) could be assigned as *Z* configuration [14]. Accordingly, the compound with olefinic proton resonance at *δ* 7.53 (d, *J* = 2.9) could be assigned as *E* configuration [15,16,17,18]. The resonance of alkylidene olefinic proton with an *E* configuration, displayed an approximately 1 ppm downfield shift from that of the *Z* configuration in both alkylidene and phenylidene substituents. Petromylidene B is a mixture of *Z*-(**2**) and *E*-(**2**) with approximate ratio of 3:1, as deduced by the integral area in the ^1^H NMR spectrum. Therefore, the structures of **2** were assigned as 2-(*Z*)-benzylidene-7*α*,12*α*,24-trihydroxy-5*α*-cholan-3-one-24-sulfate and 2-(*E*)-benzylidene-7*α*,12*α*,24-trihydroxy-5*α*-cholan-3-one-24-sulfate, trivially named (*Z*)- and (*E*)-petromylidene B, respectively.

Petromylidene C (**3**) was obtained as a pale-colored oil with [α]${}_{D}^{25}$ −22.8 (*c* 0.10, MeOH). Its molecular formula, C_26_H_42_O_7_S with *m*/*z* 497.2553 [M−H]^−^ (Supplementary Materials Figure S3), indicated six degrees of unsaturation. Its ^1^H NMR and ^13^C NMR data (Table 1, Supplementary Materials Figures S16 and S17) were similar to those of **1** and **2**. The 3kPZS (**4**), as well as the steroidal scaffold and the hydroxyl stereochemistry, was assigned using the strategies described for HMBC correlations from H-1′ to C-1 and C-3, and from H-1 to C-1′. The comparison of the olefinic proton resonance of **3** with those of (*E*)-2-ethylidenecyclohexanone [11], supported the *E* configuration for the double bond between C-2 and C-1′. The 3 exists only as 2E isomer, since no proton resonance was observed around *δ*_H_ 5.80 ppm. Therefore, **3** was assigned as 2-(*E*)-ethylidene-7*α*,12*α*,24-trihydroxy-5*α*-cholan-3-one-24-sulfate and was trivially named petromylidene C.

**Figure 4:**
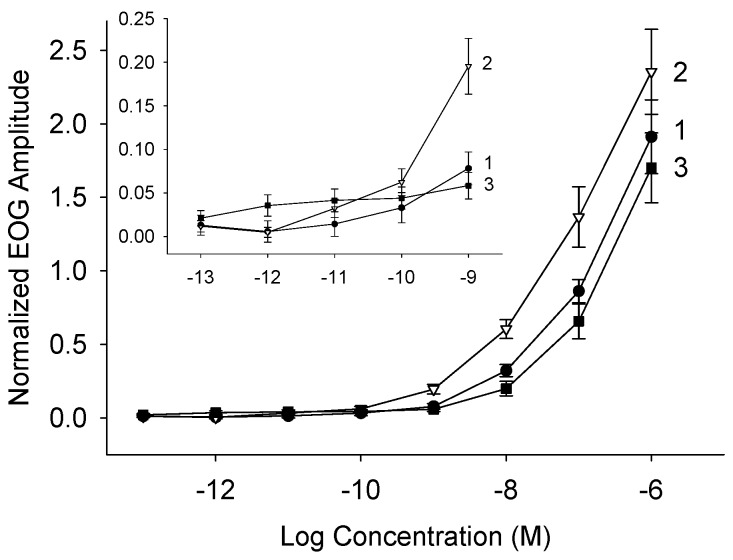
Semi-logarithmic plot of electro-olfactogram (EOG) concentration response curves show **1**–**3** (petromylidenes A–C, respectively) are stimulatory to the adult sea lamprey olfactory epithelium and have low detection thresholds. The numbers on the right of the figure correspond to each compound (filled circle **1**; open triangle **2**; filled square **3**). Data are presented as the mean normalized EOG amplitude (n = 7). Vertical bars represent one standard error of the mean. Insert: Expanded view of responses showing response threshold concentrations.

Petromylidenes A–C (**1**–**3**) were all potent odorants, which stimulated the adult sea lamprey olfactory epithelium in the electro-olfactogram (EOG) assays. In the EOG recordings, **2** was the most potent and elicited larger responses than the other compounds across a range of concentrations (10^−10^ M to 10^−6^ M), followed by **1**, and then **3** (Figure 4). The olfactory potencies of **1**–**3** at 10^−6^ M, were approximately one third of that of 3kPZS at 10^−6^ M [19] and were comparable to that of 3,12-diketo-4,6-petromyzonene-24-sulfate (DKPES), a previously described pheromone released by spermiated male sea lampreys, at 10^−6^ M [20]. The threshold of detection—the lowest concentration that elicited an olfactory response greater than the blank water control—for **1** was 10^−9^ M (paired *t*-test, *t* = −4.94, *df* = 6, *p* = 0.001), for **2** was 10^−11^ M (*t* = −3.53, *df* = 6, *p* = 0.006), and for **3** was less than 10^−13^ M (*t* = −2.52, *df* = 6, *p* = 0.023) (Figure 4). The olfactory responses of pheromonal bile acids released by spermiated male sea lamprey, had detection thresholds of 10^−10^ M to 10^−13^ M [7,20]. The threshold of detection of 3kPZS is 10^−12^ M [19].

**Figure 5:**
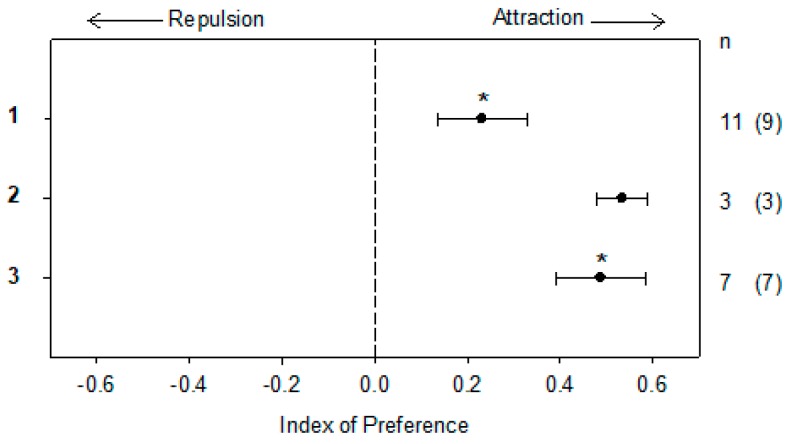
Ovulated female sea lampreys were attracted to petromylidene A (**1**) and petromylidene C (**3**) in the two-choice maze (*p* < 0.05). The time the lamprey spent in the treatment or vehicle channel of the maze before and after odorant exposure, was used to calculate an index of preference (see Equation (2) in Section 3). A positive index value indicates attraction and a negative index value indicates repulsion. Data are presented as the mean ± S.E.M. and evaluated using a Wilcoxon signed-rank test. n, sample size, with the number in the parentheses indicating the number of test subjects spending more time in the treatment side.

In a two-choice maze behavioral assay, ovulated female sea lampreys were attracted to 10^−12^ M of **1** (mean index of preference ± S.E.M.; 0.232 ± 0.096, *n* = 11) and 10^−12^ M of **3** (0.488 ± 0.097, n = 7) (Wilcoxon signed-rank test, *p* < 0.05; Figure 5 and Table S1), at comparable magnitudes to that of 10^−12^ M of 3kPZS [21]. Ovulated females also appeared to be attracted to 10^−12^ M of **2** (0.534 ± 0.054, n = 3, *p* = 0.250; Figure 5 and Supplementary Materials Table S1). However, a larger sample size was not possible due to the limited quantity of **2** available for the behavioral assays.

## 3. Experimental Section

### 3.1. General Experimental Procedures

ESIMS and HRESIMS spectra were measured using a TQ-S TOF LC mass spectrometer (Waters Corporation, Milford, MA, USA). 1D- and 2D-NMR spectra were recorded on an Agilent 900 MHz spectrometer. Column chromatography was performed on silica gel (70–230 and 230–400 mesh, Merck, Darmstadt, Germany) and Sephadex LH-20 (Merck). Semi-preparative HPLC (Waters 1525 binary HPLC pump, 2996 photodiode array detector, and fraction collector III) was performed with a Luna RP-18 column (250 × 10 mm i.d.; 5 μm; Phenomenex), eluted with methanol and H_2_O with a flow rate of 3.0 mL/min at room temperature. A gradient of methanol in H_2_O from 30 to 100% over 30 min was used. Pre-coated silica gel GF254 plates (Merck) were used for TLC. Spots were first visualized under UV light at 254 nm, and then stained by spraying with an acidic methanol solution of 5% anisaldehyde (Sigma-Aldrich, St. Louis, MO, USA), and heated on a hot plate.

### 3.2. Animal Materials

All experimental procedures involving sea lampreys were approved by the Michigan State University Institutional Animal Use and Care Committee (IACUC) (Animal usage form number: 03/14-054-00). Adult sea lampreys were captured in tributaries of the Laurentian Great Lakes by the U.S. Fish and Wildlife Service and Fisheries and Oceans Canada, according to approved scientific collection permits from those government agencies, transported to the U.S. Geological Survey Hammond Bay Biological Station, Millersburg, Michigan, USA, and held in 500–1000 L aerated tanks supplied with Lake Huron water at 15–19 °C. Adult sea lampreys used for EOG recordings were transported to the University Research Containment Facility at Michigan State University, East Lansing, Michigan, USA, and were held in flow-through tanks (250 L) supplied with aerated, chilled well water, maintained at 7–9 °C.

To produce ovulated females as test subjects for the behavioral assays conducted in June and July 2016, immature female sea lampreys were transferred to acclimation cages constructed of polyurethane mesh and PVC pipe (0.5 m^3^), located in the lower Ocqueoc River, Millersburg, Michigan to allow natural maturation. Sea lampreys were monitored daily for signs of sexual maturation, including secondary sexual characteristics and expression of ovulated oocytes from the cloacal aperture, following gentle pressure to the abdomen.

### 3.3. Extraction and Isolation

The extraction method used in this study is an adaptation of a previously reported method, developed and optimized, for sea lamprey conditioned water [20,22]. Briefly, spawning phase male sea lamprey (15–30 animals) were placed in a tank and supplied with 50 L of aerated Lake Huron water maintained at 16–18 °C. The extraction of conditioned water was performed by solid phase extraction, i.e., passed through a bed of 2 kg of Amberlite XAD 7 HP resin, contained in a series of four 2.5 L-capacity glass columns (Ace Glass Inc., Vineland, NJ, USA). Load speeds were maintained between 400–600 mL·6 min^−1^. Natural products were eluted with 10 L of methanol, followed immediately by 5 L of acetone. During June and July 2014, ten batches of the male-conditioned water was extracted. The solvent from each batch was removed under reduced pressure at 40 °C by rotoevaporation (Büchi RotovaporH, Flawil, Switzerland) and combined. The water residue was concentrated by lyophilization (Labconco Corporation, KS, USA). The extracts were suspended in methanol and filtered. Filtrates were collected and concentrated under reduced pressure at 40 °C by rotoevaporation and produced 2.1 g of extract.

### 3.4. Purification of Petromylidene A-C (**1**–**3**) and 3kPZS (**4**)

The extract was subjected to liquid chromatography over silica gel (150 g; gradient elution from 95% CHCl_3_/MeOH to 100% MeOH, ca. 2.5 L total volume). Eluents were pooled into 20 fractions, as guided by thin layer chromatography (TLC) analysis. Fraction 14 was concentrated to a residue (ca. 41 mg), which was further purified using Sephadex LH-20, first on a CHCl_3_-MeOH (1:1) column, and then on a MeOH (100%) column to yield **1** (3.61 mg), **2** (0.10 mg), **3** (1.13 mg), and **4** (8.86 mg).

petromylidene A (**1**): pale oil; [α]${}_{D}^{25}$ +6.6 (*c* 0.10, MeOH), ^1^H and ^13^C NMR data, see Table 1; HRESIMS *m*/*z* 539.3058 (calcd for C_29_H_47_O_7_S, 539.3048 [M−H]^−^).

petromylidene B (**2**): pale oil; ^1^H and ^13^C NMR data, see Table 1; HRESIMS *m*/*z* 559.2729 (calcd for C_31_H_43_O_7_S, 559.2735 [M−H]^−^).

petromylidene C (**3**): pale oil; [α]${}_{D}^{25}$ −22.8 (*c* 0.10, MeOH), ^1^H and ^13^C NMR data, see Table 1; HRESIMS *m*/*z* 497.2553 (calcd for C_26_H_41_O_7_S, 497.2578 [M−H]^−^).

### 3.5. Electro-Olfactogram (EOG) Recording

Electro-olfactogram recordings were conducted following a described procedure in References [20,22], to determine the adult sea lamprey olfactory sensitivity to **1**–**3**. Sea lampreys (243.9 g ± 17.0, 502.4 mm ± 12.8; mean ± S.E.M.) were anesthetized with 3-aminobenzoic acid ethyl ester (MS222; 100 mg L^−1^; Sigma-Aldrich), immobilized with an intra-muscular injection of gallamine triethiodide (3 mg/kg of body weight, in 0.9% saline; Sigma-Aldrich), and placed in a V-shaped Plexiglas stand. Gills were continuously irrigated with aerated water containing 50 mg L^−1^ MS222, throughout the experiment. The olfactory lamellae were surgically exposed by removing the skin on the surface of the olfactory capsule. A small capillary tube delivered the stimuli and charcoal filtered water to the olfactory epithelium by gravity flow, to prevent desiccation. The differential EOG response to each test stimulus was recorded using borosilicate electrodes filled with 0.04% agar in 0.9% saline, connected to solid state electrodes with Ag/AgCl pellets (model ESP-M15N, Warner Instruments LLC, Hamden, CT, USA) in 3M KCl. The recording electrode was placed between two olfactory lamellae and adjusted to maximize the response to L-arginine standard, whilst minimizing the response to the blank control (charcoal filtered water), and the reference electrode was placed on the external skin near the naris. Electrical signals were then amplified by a NeuroLog system (model NL102, Digitimer Ltd., Hertfordshire, England, UK), filtered with a low-pass 60 Hz filter (model NL125, Digitimer Ltd.), digitized by Digidata 1440A (Molecular Devices LLC, Sunnyvale, CA, USA), and recorded on a PC running AxoScope 10.4 software (Molecular Devices LLC). EOG recordings were conducted in April and May 2016.

For the concentration-response recordings, the olfactory epithelia of sea lampreys were exposed to 10^−13^ M to 10^−6^ M solutions of purified petromylidenes A–C (**1**–**3**). 10^−3^ M stock solutions (in 50% MeOH: deionized water) were prepared for each compound, stored at −20 °C, and then serially diluted with charcoal filtered water to yield 10^−13^ M to 10^−6^ M working solutions. A 10^−2^ M stock solution of L-arginine (in deionized water) was prepared, stored at 4 °C, and then diluted with charcoal filtered water to yield a 10^−5^ M working solution. A 10^−5^ M L-arginine standard was introduced to the olfactory epithelium for 4 s, and the olfactory response was recorded as a reference. Then, the olfactory epithelium was flushed with charcoal filtered water for 2 min. Subsequently, the blank control (charcoal filtered water) was introduced to the olfactory epithelium and recorded to confirm the absence of a response in the charcoal filtered water supply. Next, increasing concentrations of the test stimulus, starting at 10^−13^ M to 10^−6^ M, was applied to the olfactory epithelium, recorded, and flushed. Blank control and 10^−5^ M L-arginine standard was measured after every two concentrations. The EOG response magnitudes were measured in millivolts, normalized relative to that of 10^−5^ M L-arginine (2.892 ± 0.061; absolute raw value mean ± S.E.M.), and blank-corrected (0.131 ± 0.005; absolute raw value mean ± S.E.M.) as defined in Equation (1):

(1) $$\mathrm{Normalized}\;\mathrm{EOG}\;\mathrm{Amplitude}\;=\frac{\mathrm{Rt}-\mathrm{Rb}}{\mathrm{Ra}-\mathrm{Rb}}$$

where Rt is the response magnitude to the test stimulus, Rb is the response magnitude to the blank, and Ra is the response magnitude to L-arginine at 10^−5^ M. The L-arginine standard and blank control responses were comparable to previous studies [7,20,23]. The detection threshold was defined as the lowest concentration, where the test stimulus elicited a larger response than the blank control (paired *t*-test, one tailed).

### 3.6. Behavioral Assay

The behavioral responses of ovulated female sea lampreys to purified petromylidenes A–C (**1**–**3**) at 10^−12^ M were evaluated using a two-choice maze, previously described (Supplementary Materials Figure S22) in Reference [24]. A single lamprey was placed in a release cage at the furthest point downstream in the maze. After 5 min of acclimation in the cage, the lamprey was released and the cumulative amount of time the lamprey spent in each channel was recorded. This time period before odorant application, was used to assess side bias. After 10 min of recording, the test stimulus was introduced to a random channel and 50% methanol control (MeOH: deionized water) was introduced to the other channel using peristaltic pumps, at constant rates of 200 ± 5 mL min^−1^ (Masterflex 07557-00, Cole-Parmer, Vernon Hills, IL, USA). The odorants were pumped into the maze for 5 min without recording the lamprey’s behavior. After the 5 min period, the behavior was recorded for an additional 10 min, whilst odorants were continuously administered. The maze was flushed with water for 10 min, before the start of the next experiment. A 10-min flushing period was deemed to be sufficient time in previous experiments [9] and was confirmed with a rhodamine dye test.

An index of preference was calculated for each trial as defined in Equation (2):

(2) $$\mathrm{Index}\;\mathrm{of}\;\mathrm{preference}=\frac{\mathrm{Ae}\;}{\left(\mathrm{Ae}+\mathrm{Be}\right)}-\frac{\mathrm{Ac}}{\left(\mathrm{Ac}+\mathrm{Bc}\right)}$$

where Bc is the time spent by the test animal in the control channel before odorant application, Be is the time spent in the experimental channel before odorant application, Ac is the time spent in the control channel after odorant application, and Ae is the time spent in the experimental channel after odorant application. The index results in a single number, which can be either positive or negative. The indices of preference were evaluated using a Wilcoxon signed-rank test (*α* = 0.05), to determine if the index of preference was significantly different from zero. A significant positive value of the index of preference, indicated attraction. A significant negative value of the index of preference, indicated repulsion. A non-significant value of the index of preference, indicated neutral. For the statistical analysis, each animal tested was considered to be one independent data point. The trial was discarded if the sea lamprey failed to enter the control and experimental channel for at least 10 s during the 10 min period before the odorant was applied, as this was an indication of strong side bias.

## 4. Conclusions

Three previously unreported bile salts were identified from water conditioned with sexually mature male sea lampreys. Compounds **1**–**3** are highly similar in their configuration and substitution pattern, including all *trans*-fused rings, a 5*α*-H typical of fully saturated rings, and a sulfated ester on the terminal carbon of the steroidal sidechain. Together with 5*α*-myxinol-3*β*,27-disulfate found in hagfish [8] and 5*α*-cyprinol-27-sulfate found in Cypriniformes [6], **1**–**3** highlight the unique structural diversity of the 5*α* bile salt family. The alkylidene substituents have the *E* configuration for **1** and **3**. The benzylidene substituent exists in both *E* and *Z* configurations for **2**. The *Z* configuration was preferred, compared to the *E* configuration (*Z* to *E* ratio = 3:1). The oxo and hydroxyl groups preferentially occupied C-3. Notably, **1**–**3** are potent odorants that elicit attractive behavioral responses in the adult sea lampreys, indicating they possibly function as pheromones. Continued research on **1**–**3** and all sea lamprey pheromone components may lead to novel tools with which to control the invasive populations, and provide unique insights to the evolution of multi-component pheromones in vertebrates [25].

## Figures and Tables

**Table 1:** NMR Spectroscopic Data (900 and 225 MHz, MeOD) for petromylidenes A, B, and C (**1**–**3**).

No	Petromylidene A (1)		Petromylidene B (2)		Petromylidene C (3)	
	*δ*_H_ (*J* in Hz)	*δ*_C,_ Type	*δ*_H_ (*J* in Hz)	*δ*_C,_ Type	*δ*_H_ (*J* in Hz)	*δ*_C,_ Type
1*α*	1.93, d (16.1)	40.9, CH_2_	2.28, m	51.3, CH_2_	1.95, d (15.6)	40.5, CH_2_
1*β*	2.77, d (16.1)		2.64, d (13.6)		2.77, d (15.4)	
2		137.3, C		140.4, C		137.6, C
3		203.4, C		207.7, C		203.2, C
4*α*	2.15, m	43.6, CH_2_	2.18, dd (14.9, 4.1)	47.6, CH_2_	2.15, m	43.5, CH_2_
4*β*	2.22, m		2.52, m		2.22, m	
5	2.22, m	37.0, CH	2.27, m	41.1, CH	2.22, m	37.0, CH
6*α*	1.47, m	37.5, CH_2_	1.52, m	37.7, CH_2_	1.47, m	37.5, CH_2_
6*β*	1.55, m		1.58, m		1.55, m	
7*β*	3.81, ddd (7.8, 2.6, 2.6)	68.1, CH	3.80, dd (2.5, 5.3)	68.3, CH	3.81, ddd (2.5, 5.2)	68.1, CH
8*β*	1.46, m	41.3, CH	1.49, m	41.4, CH	1.46, m	41.4, CH
9*α*	1.82, ddd (12.5, 4.6, 2.0)	39.6, CH	1.82, m	39.8, CH	1.83, m	39.7, CH
10		36.5, C		39.2, C		36.4, C
11*α*	1.68, m	29.8, CH_2_	1.72, m	30.2, CH_2_	1.70, m	30.0, CH_2_
11*β*	1.72, m					
12	4.00, dd (2.6, 2.6)	73.9, CH	4.01, dd (2.9, 2.9)	73.9, CH	4.02, dd (2.6, 2.6)	73.9, CH
13		47.6, C		47.7, C		47.6, C
14*α*	1.99, ddd (7.5, 12.0, 12.2)	43.2, CH	2.00, ddd (7.6, 12.0, 12.2)	43.2, CH	1.99, ddd (7.5, 12.0, 12.2)	43.2, CH
15*α*	1.13, m	24.3, CH_2_	1.13, m	24.3, CH_2_	1.14, m	24.3, CH_2_
15*β*	1.78, m		1.78, m		1.78, m	
16*α*	1.31, m	28.9, CH_2_	1.30, m	28.9, CH_2_	1.31, m	28.9, CH_2_
16*β*	1.91, m		1.88, m		1.91, m	
17*α*	1.86, ddd (9.5, 8.9, 9.0)	48.4, CH	1.86, ddd (8.8, 8.8, 9.0)	48.4, CH	1.86, ddd (9.5, 8.9, 9.0)	48.4, CH
18	0.75, s	13.2, CH_3_	0.76, s	13.2, CH_3_	0.75, s	13.2, CH_3_
19	0.81, s	11.2, CH_3_	0.81, s	11.3, CH_3_	0.81, s	11.1, CH_3_
20	1.39, m	36.8, CH	1.40, m	37.1, CH	1.40, m	36.8, CH
21	1.04, d (6.6)	18.1, CH_3_	1.04, d (6.6)	18.1, CH_3_	1.04, d (6.6)	18.1, CH_3_
22a	1.16, m	33.3, CH_2_	1.16, m	33.3, CH_2_	1.16, m	33.3, CH_2_
22b	1.54, m		1.54, m		1.54, m	
23a	1.58, m	27.4, CH_2_	1.56, m	27.4, CH_2_	1.56, m	27.4, CH_2_
23b	1.77, m		1.76, m		1.76, m	
24	3.97, m	69.8, CH_2_	3.96, m	69.8, CH_2_	3.96, m	69.8, CH_2_
1′	6.68, m	141.8, CH	6.47, d (1.9, *Z*)/7.53 d (2.9, *E*)	134.3 (*Z*)/138.8 (*E*), CH	6.75, m	137.7, CH
2′a	2.02, m	38.1, CH_2_		137.4, C	1.76, dd (7.2, 1.9)	13.9, CH_3_
2′b	2.06, m					
3′	1.78, m	30.0, CH	7.30, m	130.0, CH		
4′	0.95, d (7.0) *	23.0, CH_3_	7.24, m	129.2, CH		
5′	0.94, d (7.0) *	23.0, CH_3_	7.44, m	131.6, CH		

* Assignments of these protons may be interchanged.

## Supplementary Materials

^1^H NMR, ^13^C NMR, COSY, HSQC, HMBC, and NOESY spectra and HRESIMS data for compounds **1**–**3** are available online at http://www.mdpi.com/1660-3397/16/9/308/s1.
