# Supplementary material for: Petromylidenes A–C: 2-Alkylidene Bile Salt Derivatives Isolated from Sea Lamprey (Petromyzon marinus)
Source: Mar Drugs. 2018 Sep 1;16(9):308. doi: 10.3390/md16090308 (PMC6163752; doi:10.3390/md16090308)
Supplement: Supplementary file 1 [file marinedrugs-16-00308-s001.pdf]

marine drugs

Supplementary Materials

Petromylidenes A–C: 2-Alkylidene Bile Salt Derivatives Isolated from Sea Lamprey (*Petromyzon Marinus*)

Ke Li, Anne M. Scott, Skye D. Fissette, Tyler J. Buchinger, Joseph J. Riedy and Weiming Li *

Department of Fisheries and Wildlife, Michigan State University, East Lansing, MI 48824, USA; like4@msu.edu (K.L.); Scottan7@msu.edu (A.M.S.); fissette@msu.edu (S.D.F.); buching6@msu.edu (T.J.B.); riedyjos@msu.edu (J.J.R.)

* Correspondence: liweim@msu.edu; Tel.: +1-517-432-6705

Table of Contents

Figure S1. HR-ESI-MS of 1 on negative mode 2
Figure S2. HR-ESI-MS of 2 on negative mode 2
Figure S3. HR-ESI-MS of 3 on negative mode 2
Figure S4. ^{1}H NMR (900 MHz) spectrum of petromylidene A (1) 3
Figure S5. ^{13}C NMR (225 MHz) spectrum petromylidene A (1) 4
Figure S6. COSY spectrum of petromylidene A (1) 5
Figure S7. HSQC spectrum of petromylidene A (1) 6
Figure S8. HMBC spectrum of petromylidene A (1) 7
Figure S9. NOESY spectrum of petromylidene A (1) 8
Figure S10. ^{1}H NMR (900 MHz) spectrum of petromylidene B (2) 9
Figure S11. ^{13}C NMR (225 MHz) spectrum petromylidene B (2) 10
Figure S12. COSY spectrum of petromylidene B (2) 11
Figure S13. HSQC spectrum of petromylidene B (2) 12
Figure S14. HMBC spectrum of petromylidene B (2) 13
Figure S15. NOESY spectrum of petromylidene B (2) 14
Figure S16. ^{1}H NMR (900 MHz) spectrum of petromylidene C (3) 15
Figure S17. ^{13}C NMR (225 MHz) spectrum petromylidene C (3) 16
Figure S18. COSY spectrum of petromylidene C (3) 17
Figure S19. HSQC spectrum of petromylidene C (3) 18
Figure S20. HMBC spectrum of petromylidene C (3) 19
Figure S21. NOESY spectrum of petromylidene C (3) 20
Figure S22. Schematic of the two-choice maze used to evaluate behavioral responses of ovulated female sea lampreys to odorants 21
Table S1. Calculated behavioral index of preference of ovulated female sea lampreys to petromylidene A (1), B (2), and C (3) as evaluated using a two-choice maze as shown in Figure S1 21

Single Mass Analysis
Tolerance = 5.0 PPM / DBE: min = -1.5, max = 50.0
Element prediction: Off
Monoisotopic Mass, Even Electron Ions
68 formula(e) evaluated with 1 results within limits (up to 50 closest results for each mass)
Elements Used:
C: 0-30 H: 0-100 O: 0-20 S: 1-1

Mass	Calc. Mass	mDa	PPM	DBE	Formula	C	H	O	S
539.3058	539.3042	1.6	3.0	6.5	C29 H47 O7 S	29	47	7	1

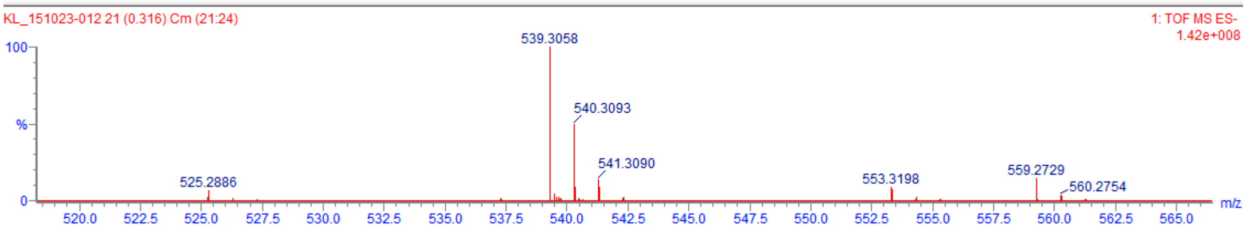

Figure S1. HR-ESI-MS of 1 on negative mode.

Single Mass Analysis
Tolerance = 5.0 PPM / DBE: min = -1.5, max = 50.0
Element prediction: Off
Monoisotopic Mass, Even Electron Ions
85 formula(e) evaluated with 1 results within limits (up to 50 closest results for each mass)
Elements Used:
C: 0-35 H: 0-100 O: 0-20 S: 1-1

Mass	Calc. Mass	mDa	PPM	DBE	Formula	C	H	O	S
559.2729	559.2729	0.0	0.0	10.5	C31 H43 O7 S	31	43	7	1

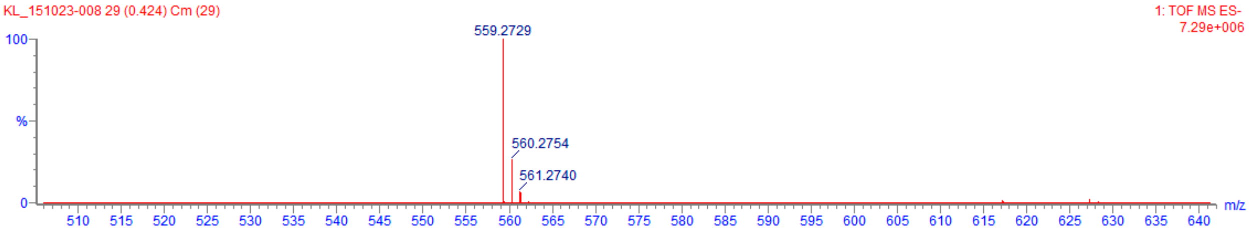

Figure S2. HR-ESI-MS of 2 on negative mode.

Single Mass Analysis
Tolerance = 5.0 PPM / DBE: min = -1.5, max = 50.0
Element prediction: Off
Monoisotopic Mass, Even Electron Ions
90 formula(e) evaluated with 1 results within limits (up to 50 closest results for each mass)
Elements Used:
C: 0-35 H: 0-100 O: 0-20 S: 1-1

Mass	Calc. Mass	mDa	PPM	DBE	Formula	C	H	O	S
497.2553	497.2573	-2.0	-4.0	6.5	C26 H41 O7 S	26	41	7	1

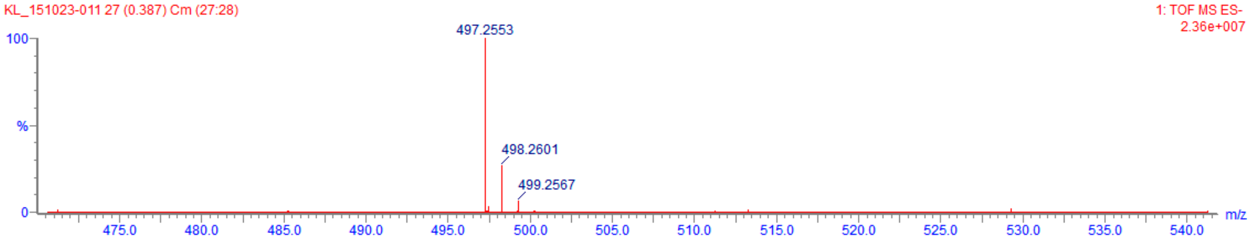

Figure S3. HR-ESI-MS of 3 on negative mode.

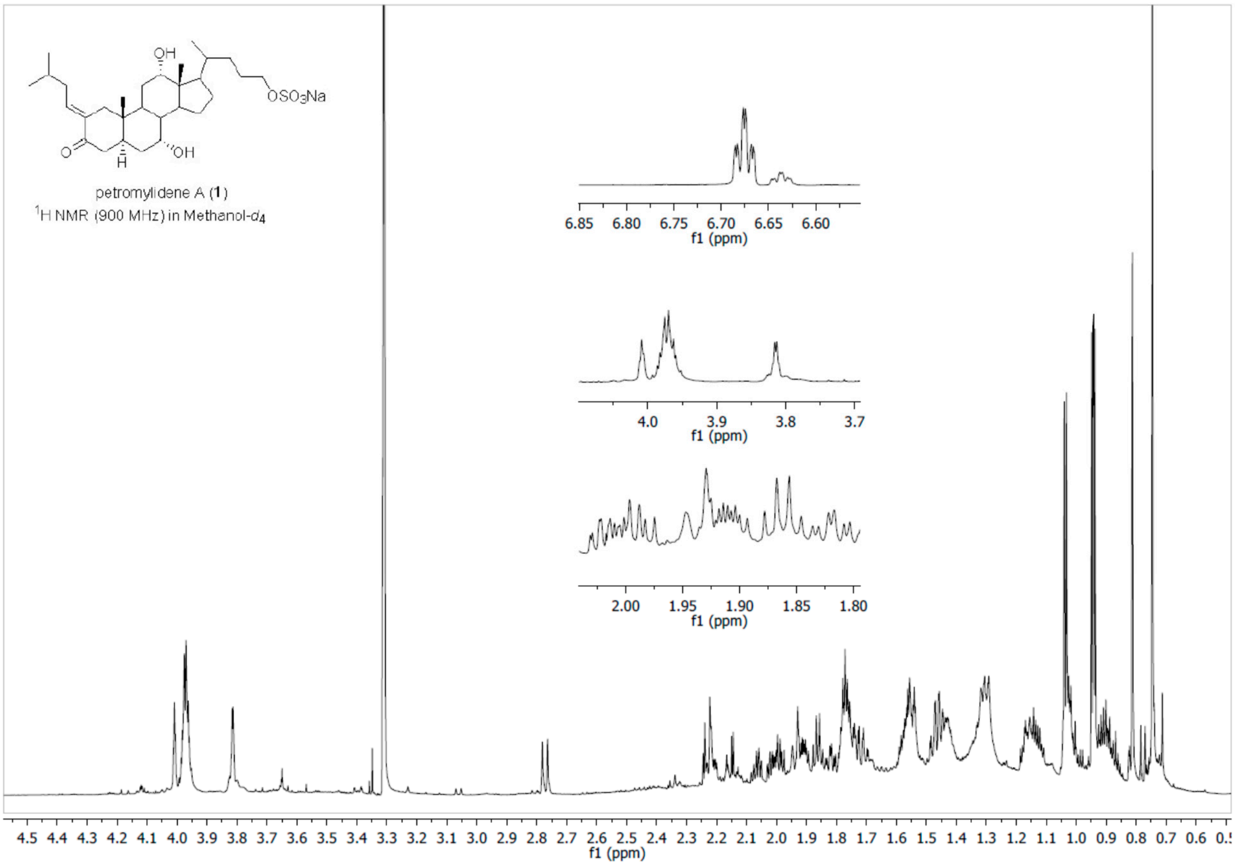

Figure S4. ^{1}H NMR (900 MHz) spectrum of petromylidene A (1).

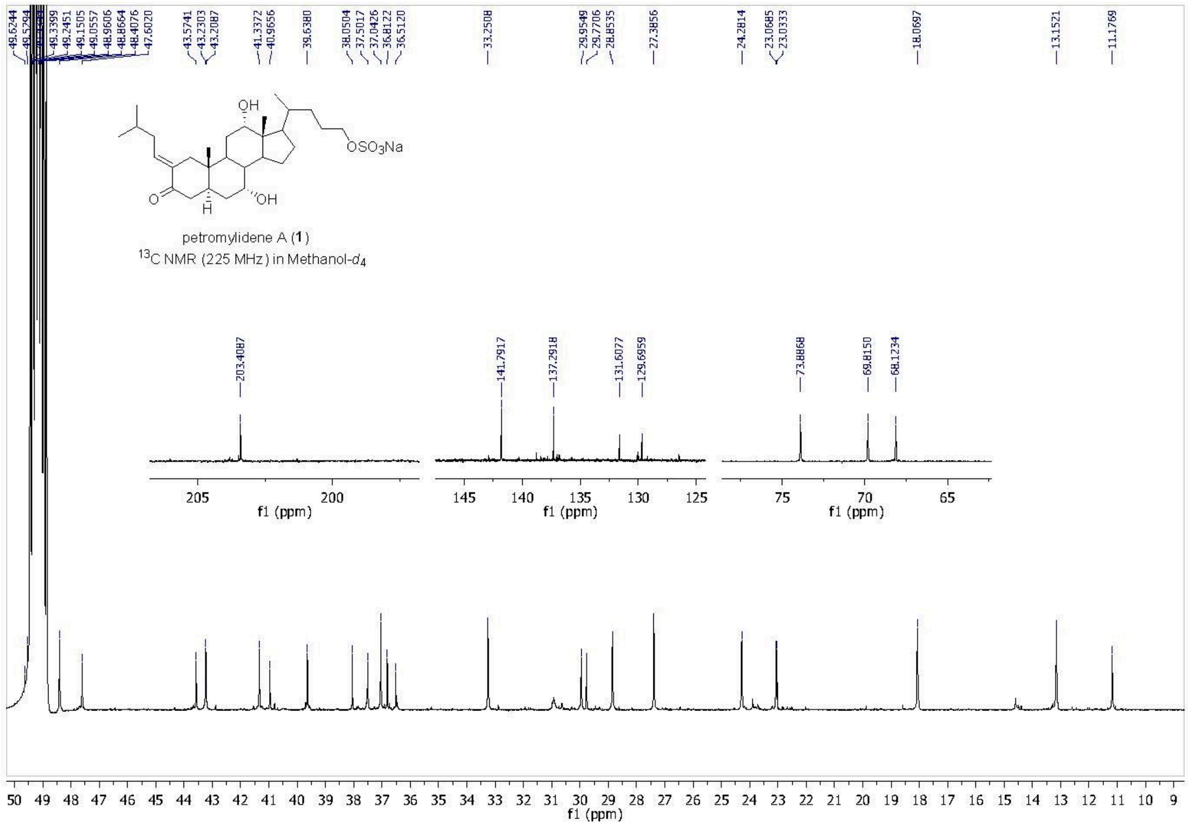

Figure S5. ^{13}C NMR (225 MHz) spectrum petromylidene A (1).

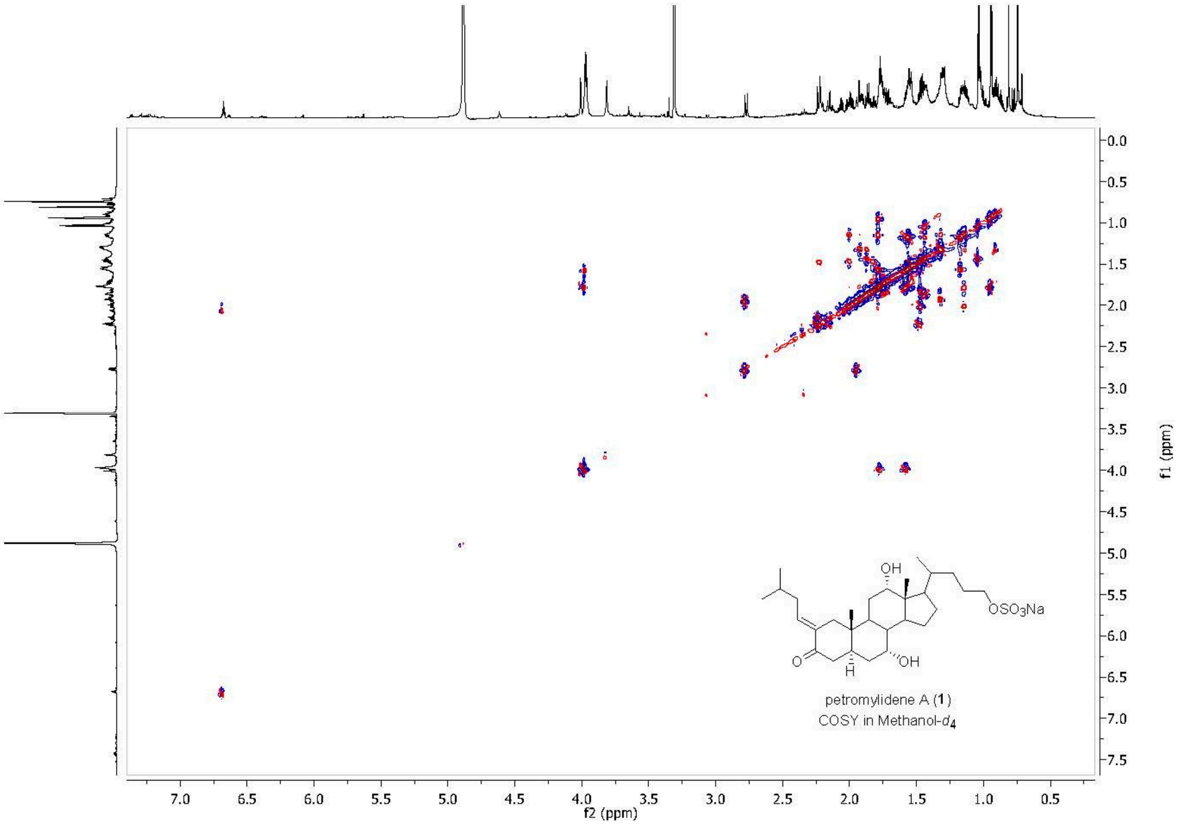

Figure S6. COSY spectrum of petromylidene A (1).

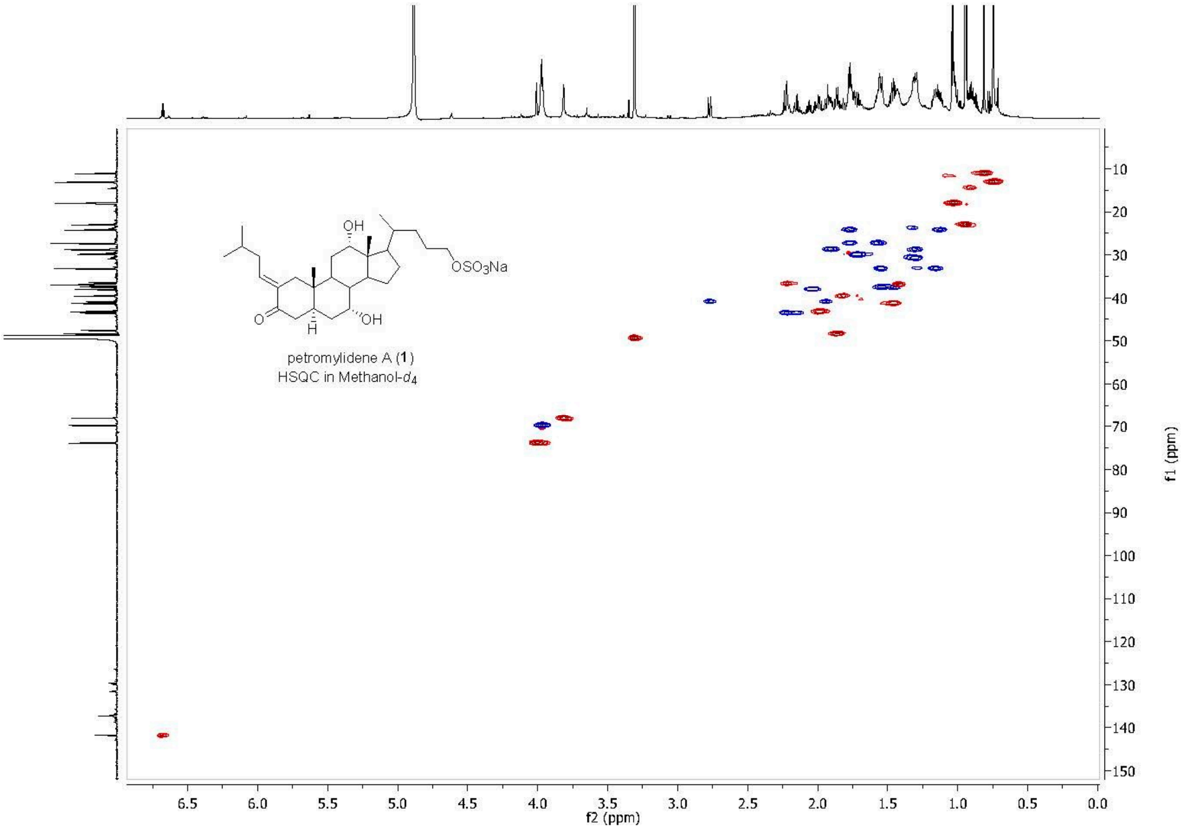

Figure S7. HSQC spectrum of petromylidene A (1).

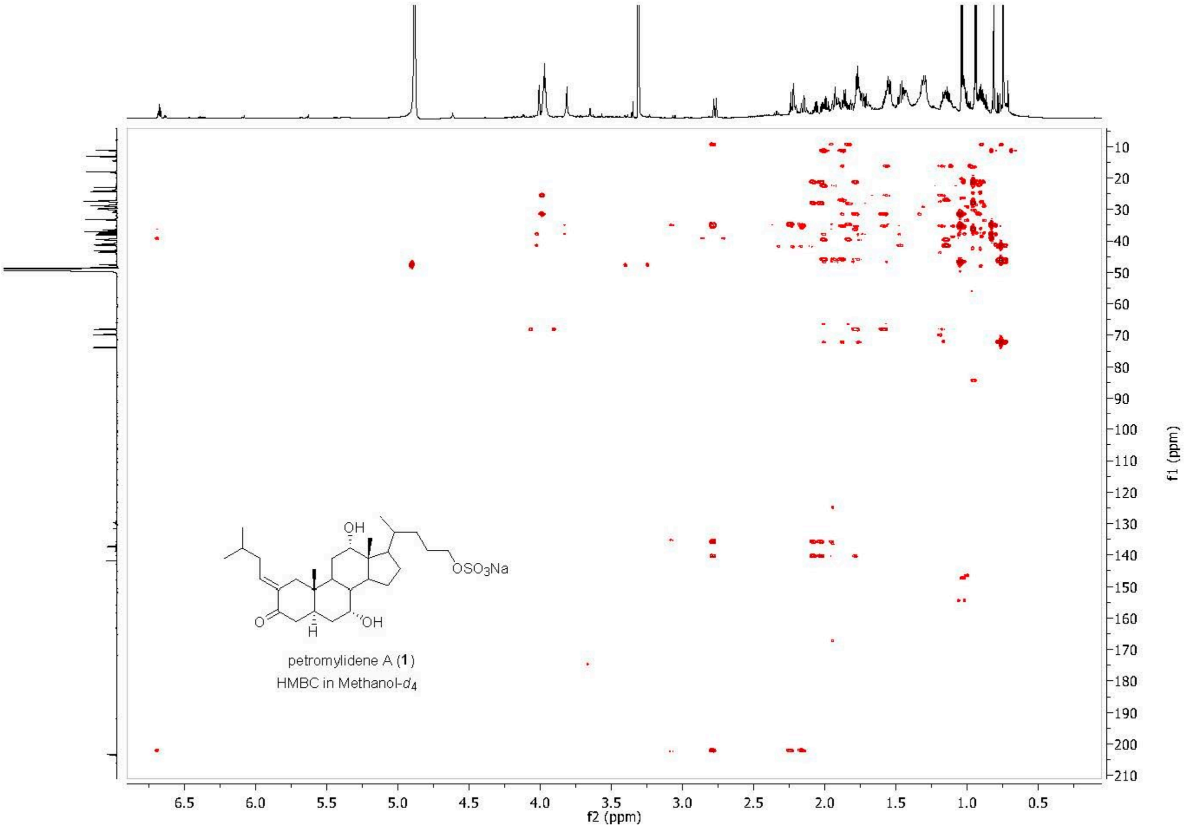

Figure S8. HMBC spectrum of petromylidene A (1).

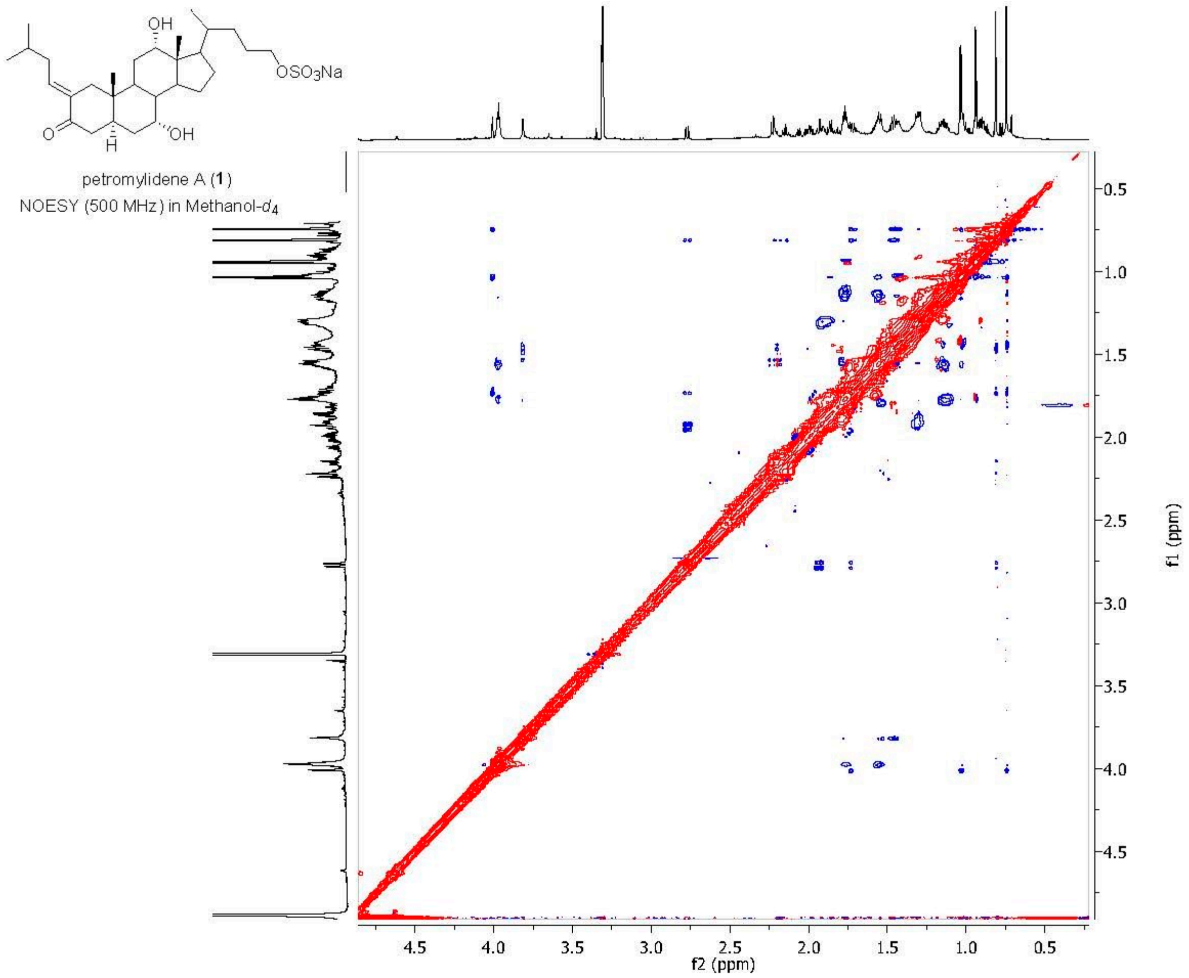

Figure S9. NOESY spectrum of petromylidene A (1).

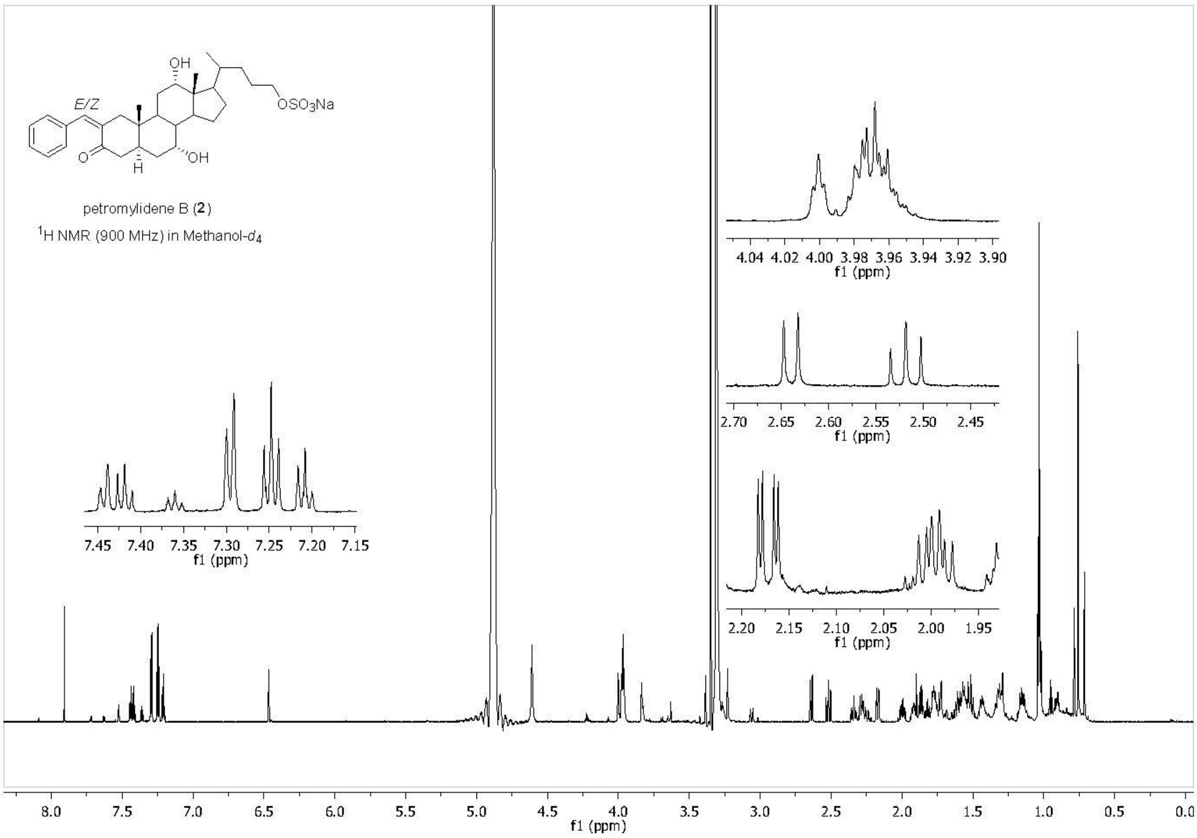

Figure S10. ^{1}H NMR (900 MHz) spectrum of petromylidene B (2).

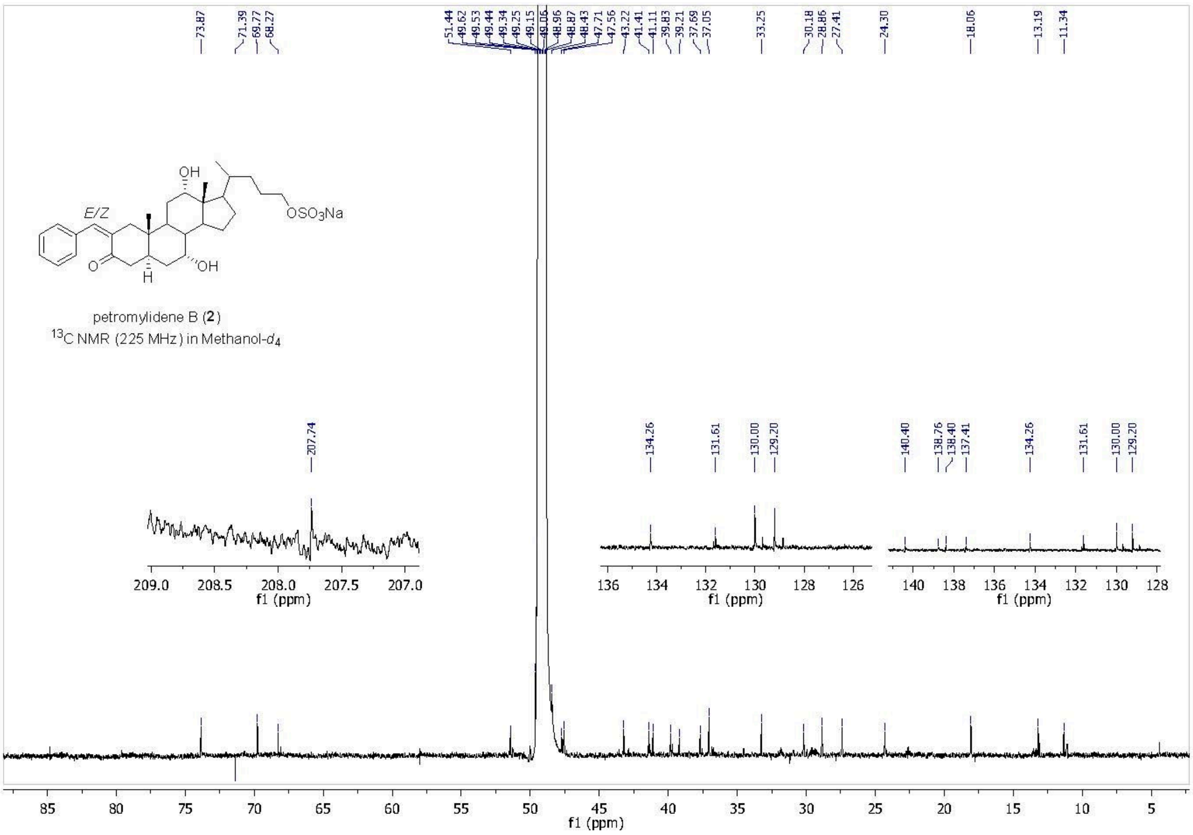

Figure S11. ^{13}C NMR (225 MHz) spectrum petromylidene B (2).

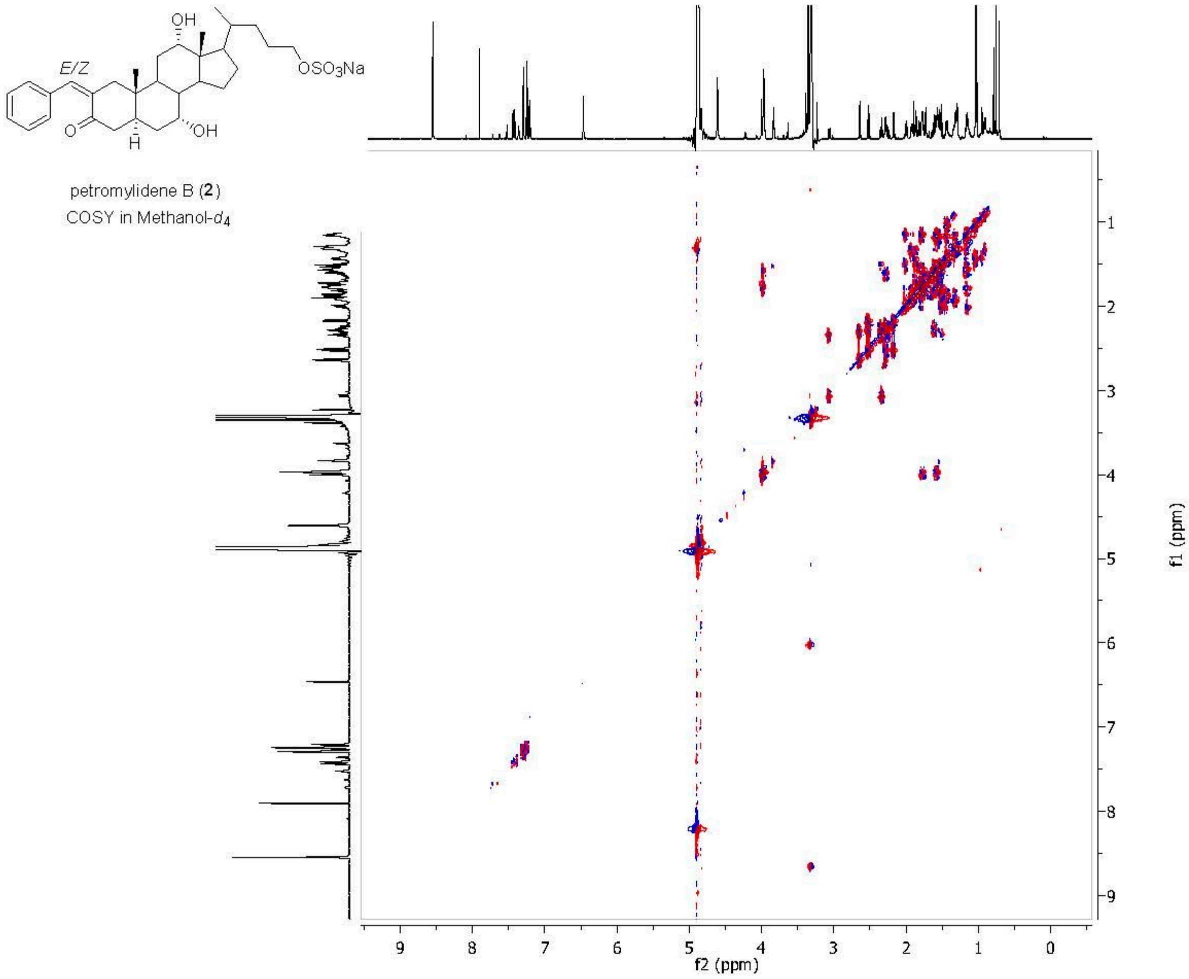

Figure S12. COSY spectrum of petromylidene B (2).

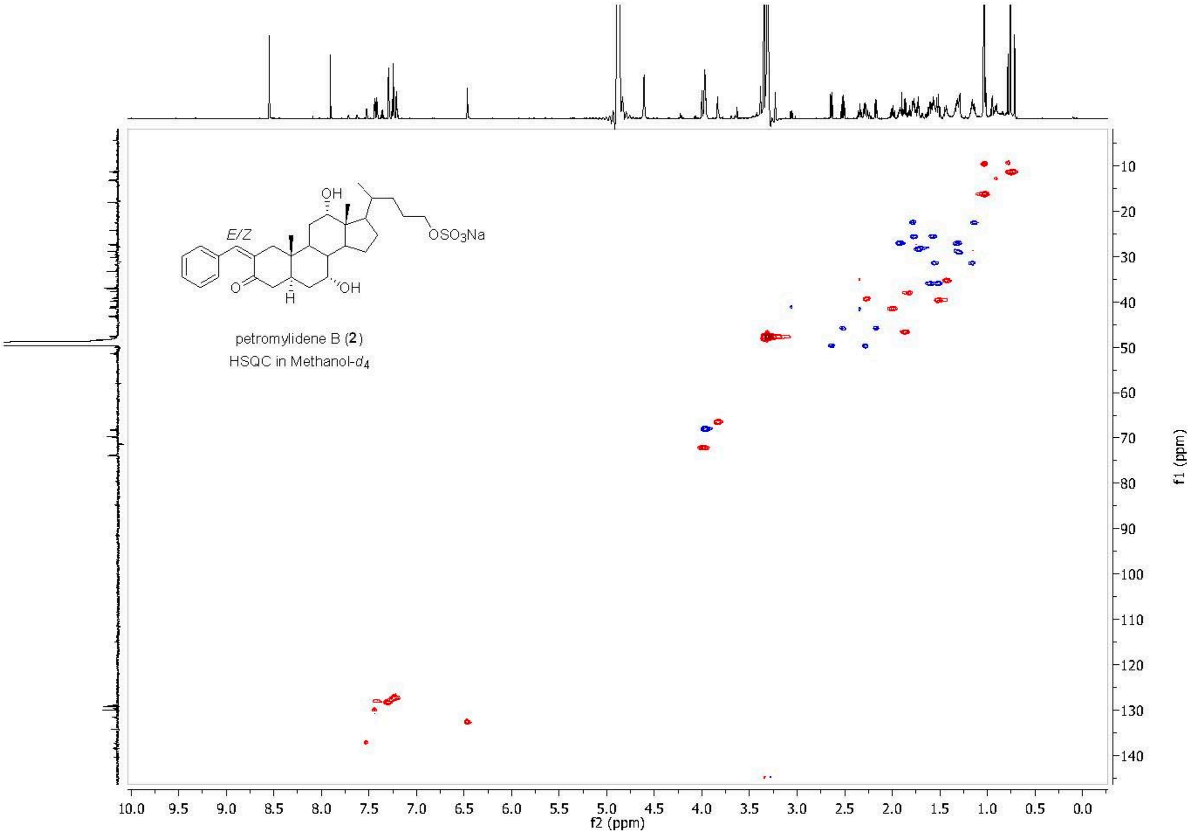

Figure S13. HSQC spectrum of petromylidene B (2).

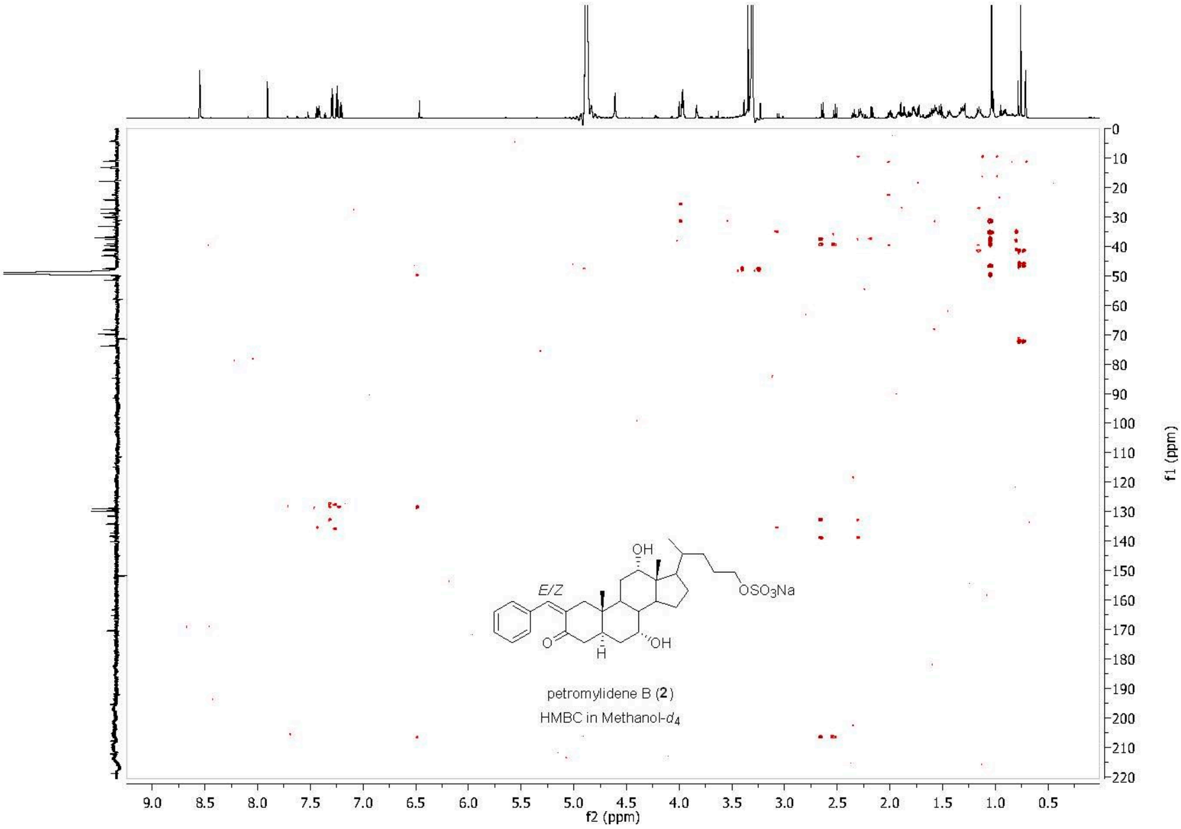

Figure S14. HMBC spectrum of petromylidene B (2).

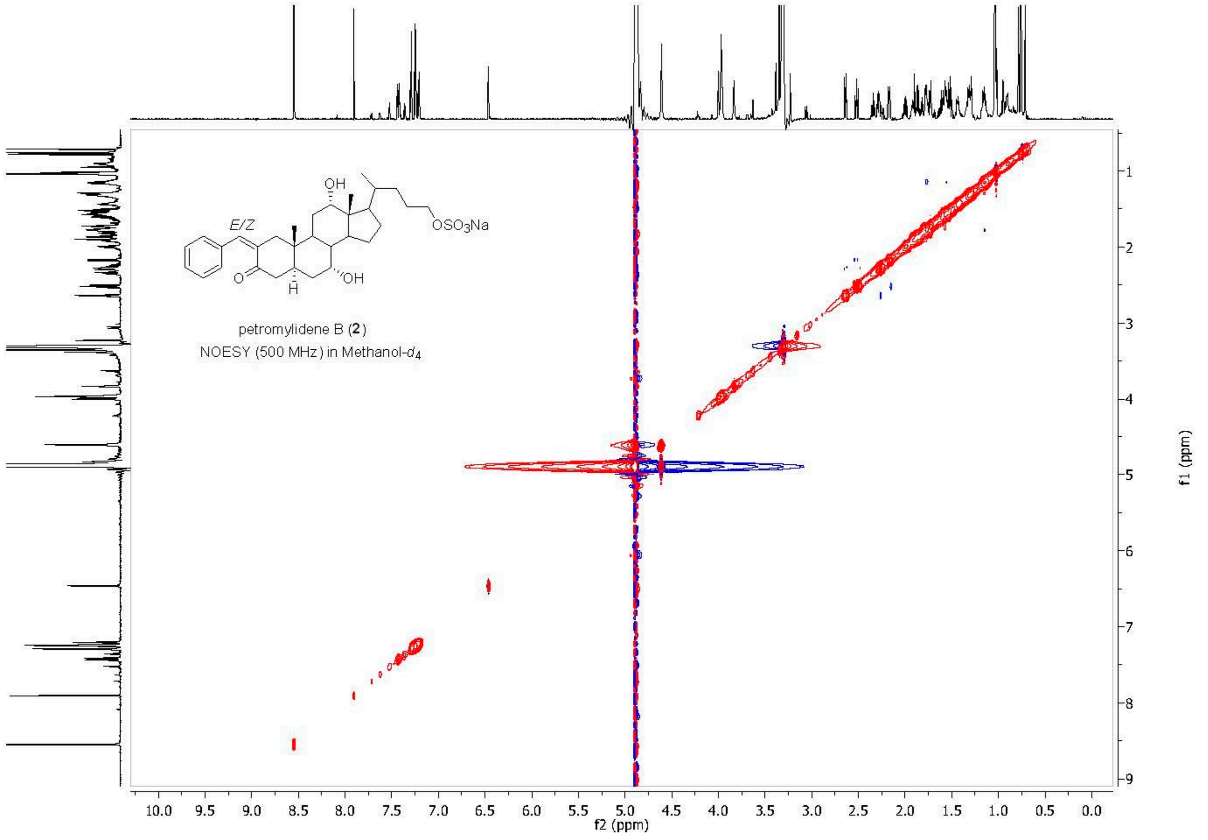

Figure S15. NOESY spectrum of petromylidene B (2).

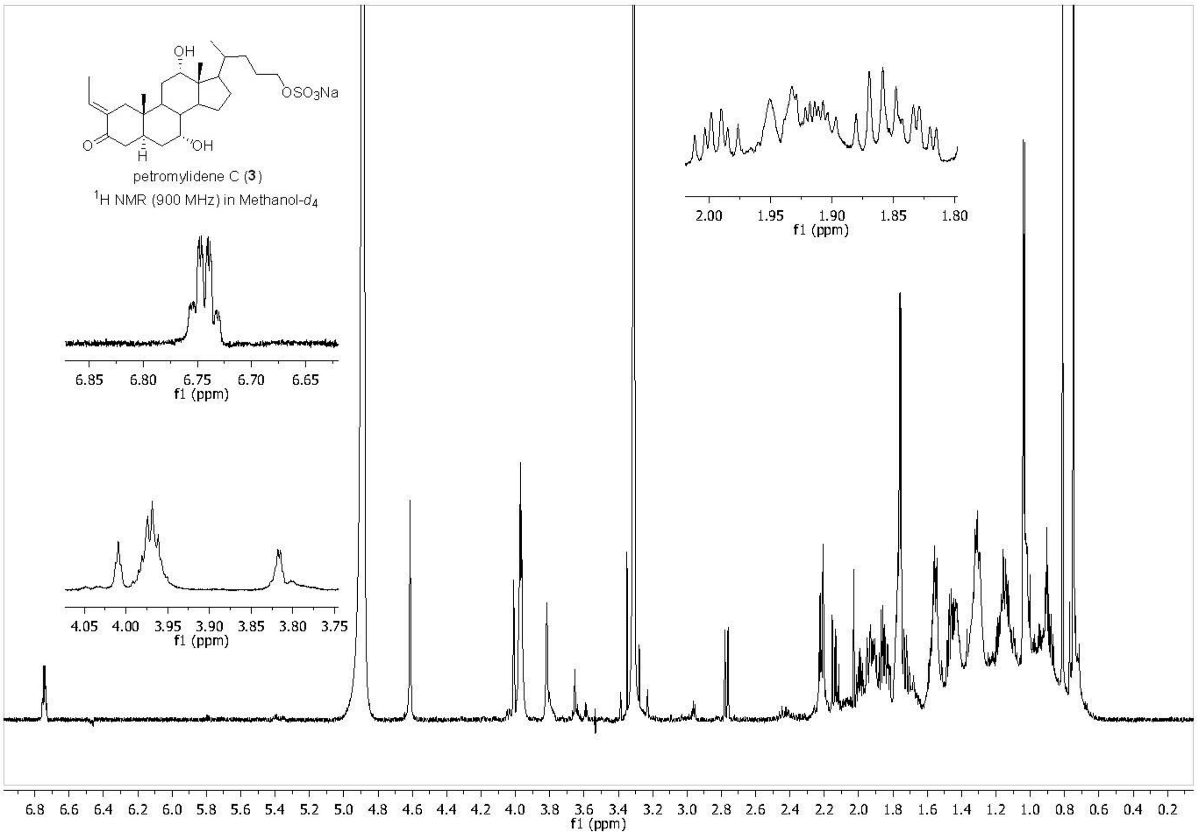

Figure S16. ^{1}H NMR (900 MHz) spectrum of petromylidene C (3).

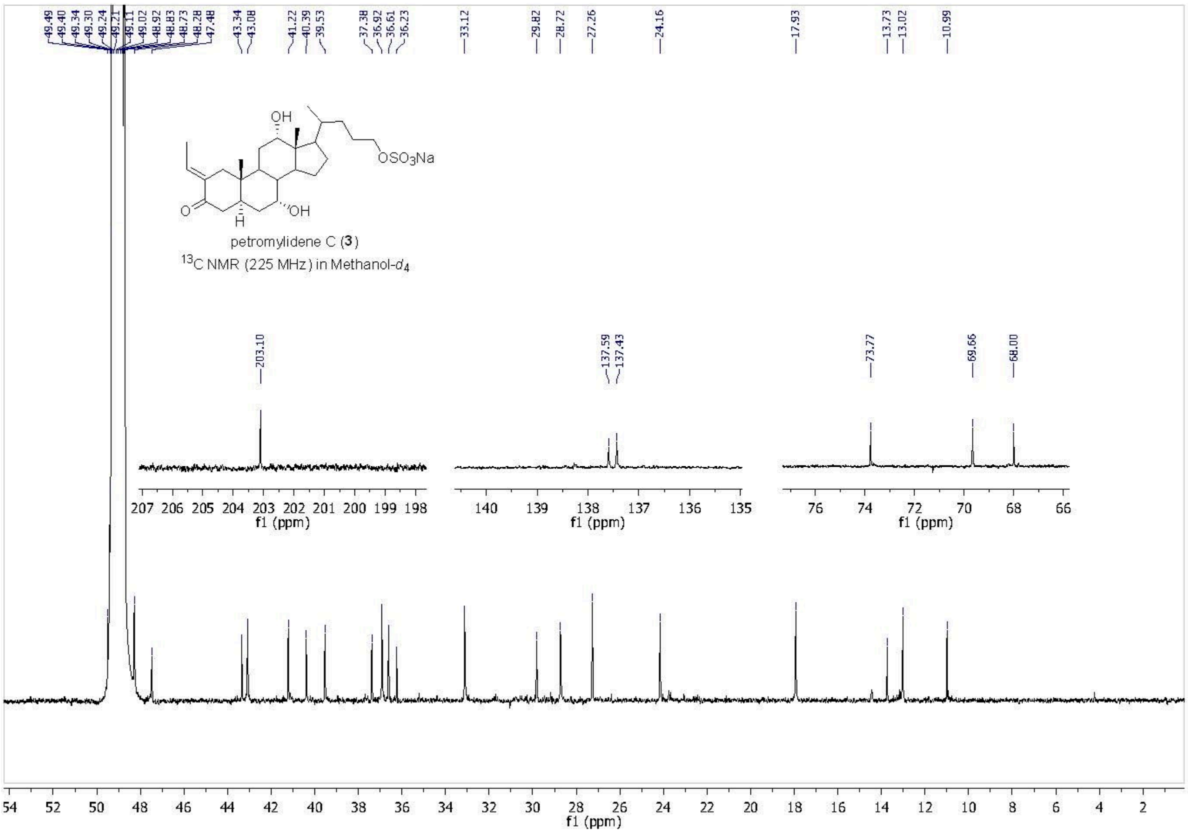

Figure S17. ^{13}C NMR (225 MHz) spectrum petromylidene C (3).

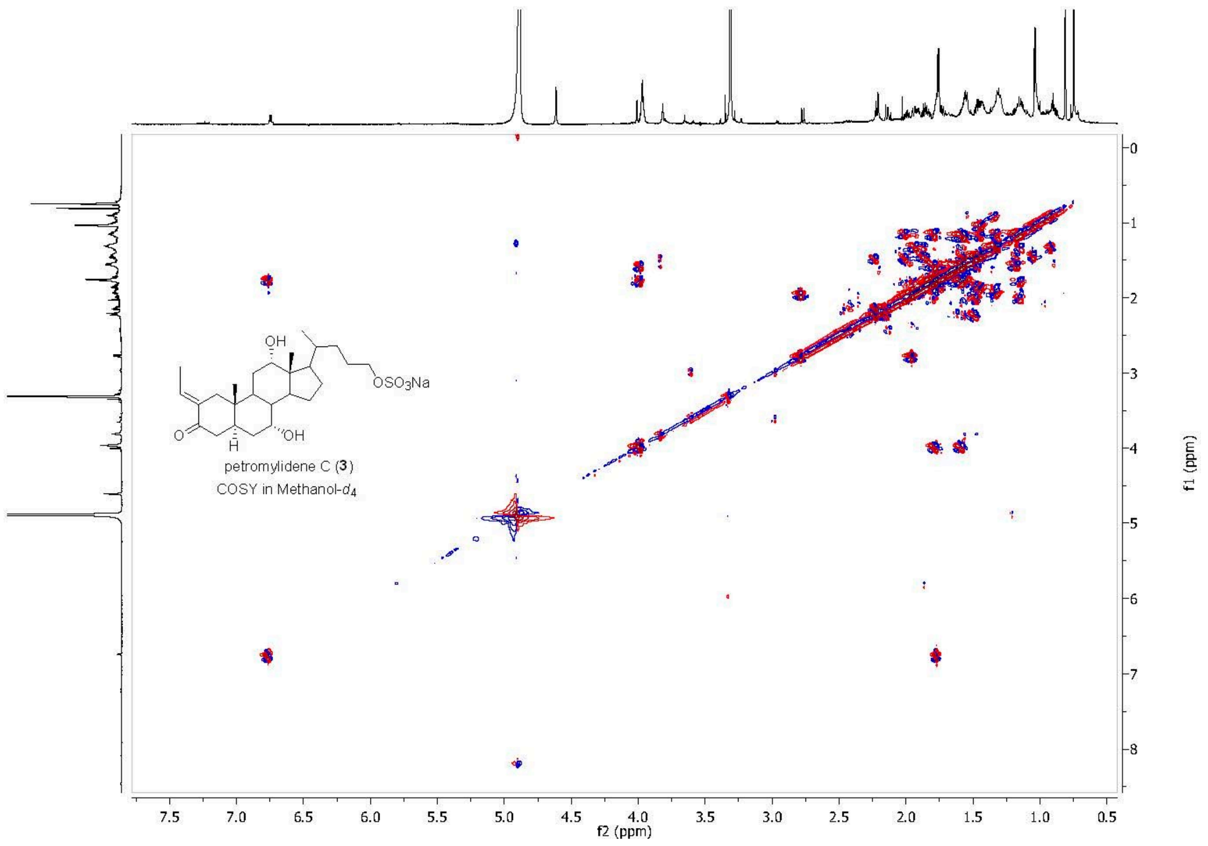

Figure S18. COSY spectrum of petromylidene C (3).

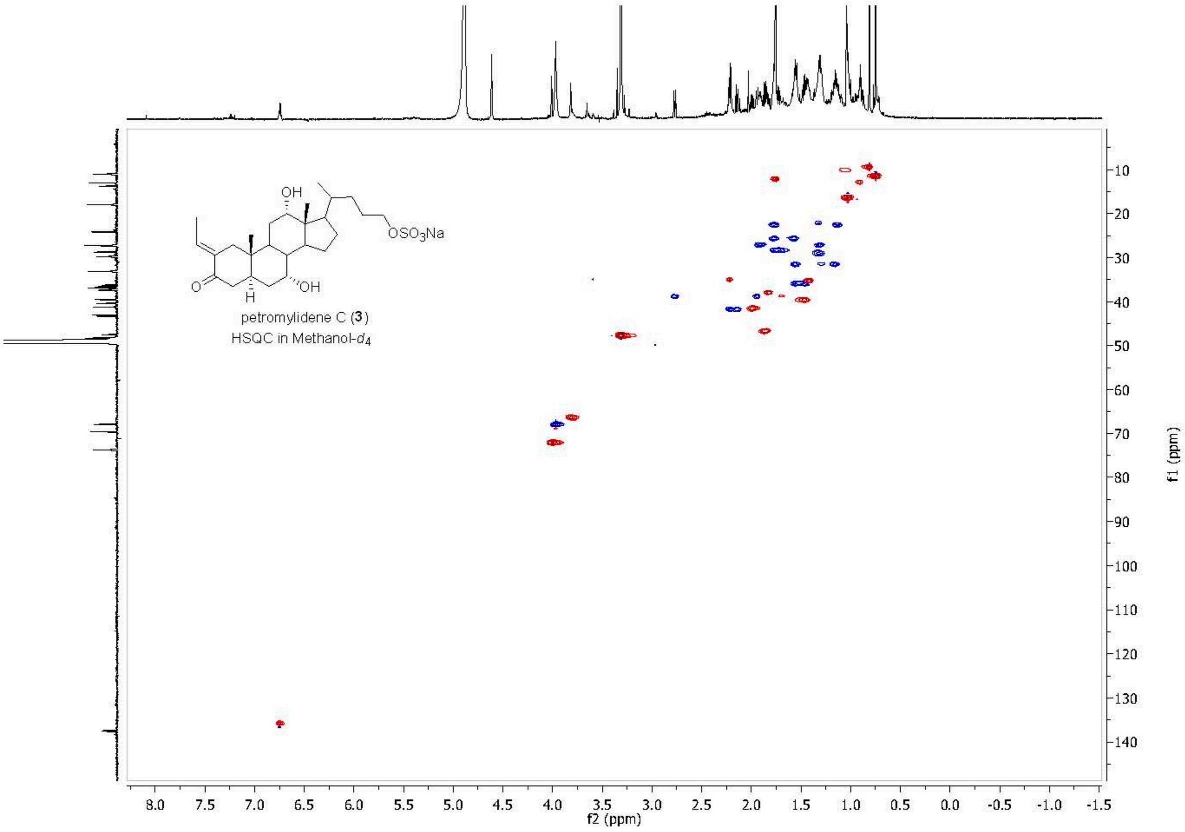

Figure S19. HSQC spectrum of petromylidene C (3).

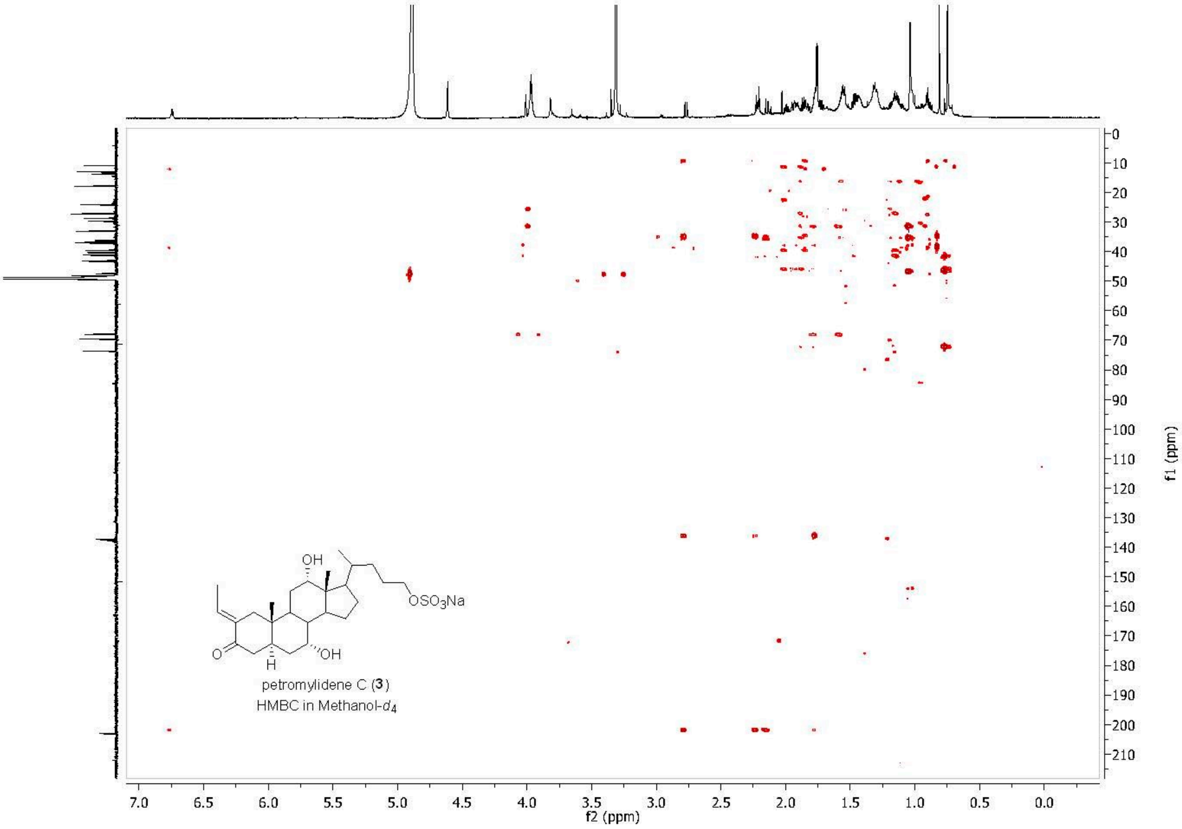

Figure S20. HMBC spectrum of petromylidene C (3).

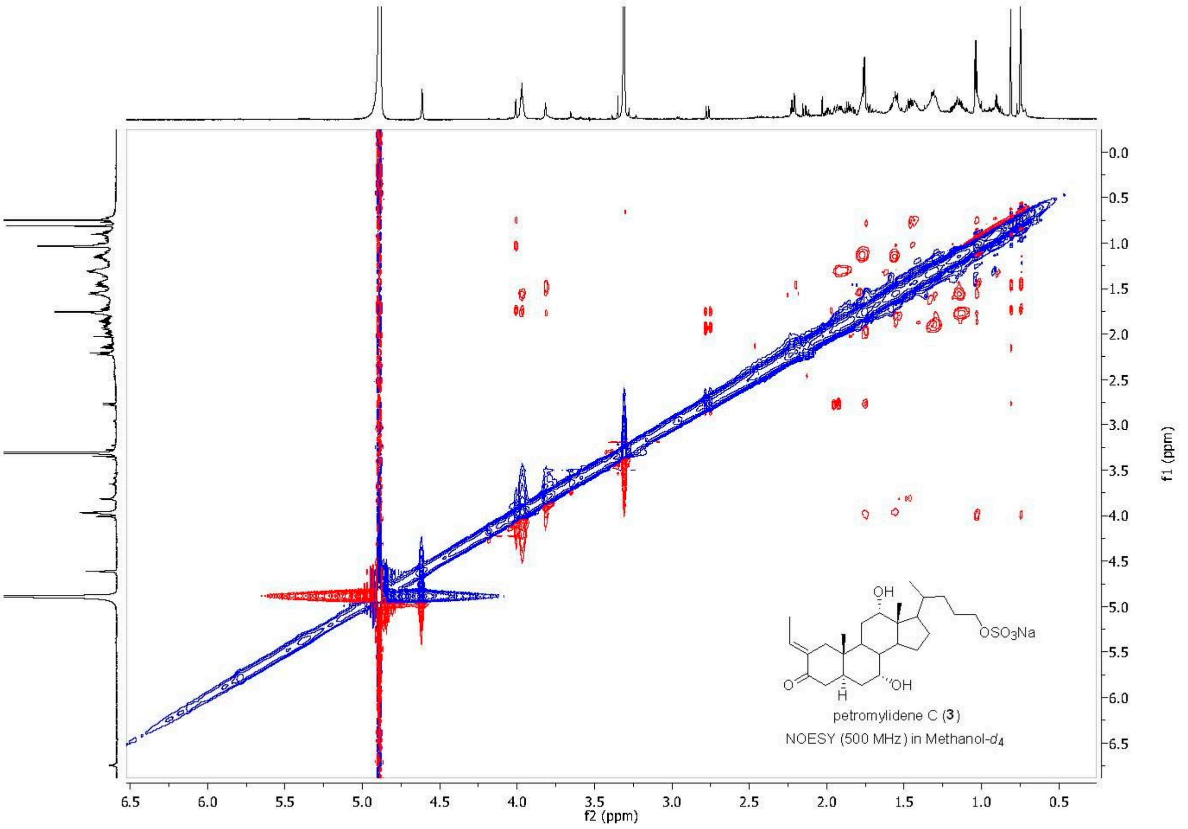

Figure S21. NOESY spectrum of petromylidene C (3).

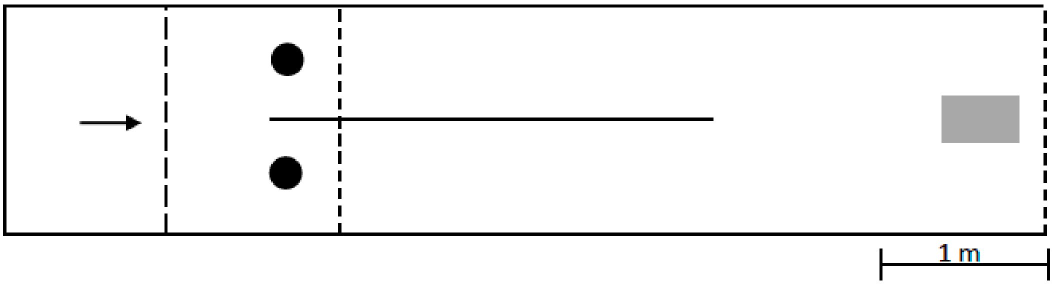

Figure S22. Schematic of the two-choice maze used to evaluate behavioral responses of ovulated female sea lampreys to odorants. The arrow represents the direction of water flow (0.07 $m \cdot s^{-1}$ ± 0.01). Circles represent odorant administration points. The gray rectangle represents the release cage. The large dashed lines represent flow boards used to reduce water turbulence. The small dashed lines represent fine mesh used to restrict the movement of the sea lampreys.

Table S1. Calculated behavioral index of preference of ovulated female sea lampreys to petromylidene A (**1**), B (**2**), and C (**3**) as evaluated using a two-choice maze as shown in Figure S1.

Odorant	n [a]	Index of Preference ± Standard Error of Mean [b]	p value [c]
Petromylidene A (1) 10^{-12} M	11	0.232 ± 0.096	0.032
Petromylidene B (2) 10^{-12} M	3	0.534 ± 0.054	0.250
Petromylidene C (3) 10^{-12} M	7	0.488 ± 0.097	0.016

[a] Number of trials. [b] See Equation 2 in the Behavioral Assay of the experimental section for details of the calculation. [c] The index of preference was evaluated using a Wilcoxon signed-rank test (α = 0.05).
